# Supplementary material for: Drosophila epidermal cells are intrinsically mechanosensitive and modulate nociceptive behavioral outputs
Source: eLife. 2025 May 12;13:RP95379. doi: 10.7554/eLife.95379 (PMC12068870; doi:10.7554/eLife.95379)
Supplement: Supplementary file 1. [file elife-95379-supp1.pdf]

Supplementary File 1: Details of Statistical Analysis

Figure 1C: Fisher's exact test with BH correction

UAS-TRPA1 groups (25 C vs 32 C)	p-value	q-value	Significance (q<0.05)
Control (no *GAL4* driver)	0.2424	2.424000e-01	F
R27H06-GAL4	2.2e-16	3.666667e-16	T
R38F11-GAL4	2.2e-16	3.666667e-16	T
R38F11-GAL4 + elav-GAL80	2.2e-16	3.666667e-16	T
R38F11-GAL4 + tshGAL80	4.111e-15	5.138750e-15	T

Figure 1S4: ANOVA with post-hoc Tukey's test

Dendrite length	p-value	Significance
R38 vs. UAS-luc RNAi	>0.9999	F
R38 vs. UAS-CsChrimson	0.9659	F
R38 vs. UAS-TRPA1	0.9982	F
R38 vs. UAS-orai RNAi	0.9009	F
R38 vs. UAS-Stim RNAi	0.9219	F
R38 vs. UAS-Stim	>0.9999	F
R38 vs. UAS-shi^TS	0.9974	F

Dendrite branchpoints	p-value	Significance
R38 vs. UAS-luc RNAi	0.9945	F
R38 vs. UAS-CsChrimson	0.9269	F
R38 vs. UAS-TRPA1	0.6859	F
R38 vs. UAS-orai RNAi	0.9896	F
R38 vs. UAS-Stim RNAi	0.9949	F
R38 vs. UAS-Stim	0.8654	F
R38 vs. UAS-shi^TS	>0.9999	F

Figure 1S6C: Fisher's exact test

	p-value	Significance
rolling	0.0002928	T
c-bend	2.035e-10	T
backing	0.0008424	T
hunch	0.004233	T
freeze	0.002342	T

Figure 1S6D: Wilcoxon rank sum test

	p-value	Significance
rolling	Cannot compare	
c-bend	0.8907	F
backing	0.00127	T
hunch	Cannot compare	
freeze	Cannot compare	

Figure 2F: Wilcoxon Rank-Sum test

	p-value	Significance
C4da vs Epi	0.0067	T

Figure 2G: Kruskal-Wallis test followed by Wilcoxon rank sum test with BH correction

	p-value	q-value	Significance (q<0.05)

Kruskal-Wallis test, rolling	N/A (only epi + C4da have values)		
rolling C4da vs Epi	0.03124		T
Kruskal-Wallis test, c-bend	0.0001652		T
c-bend C4da vs Epi	0.001468	0.002936	T
c-bend C3da vs Epi	0.3237	0.323700	F

Figure 2H Fisher's exact test with a BH correction

	p-value	q-value	Significance (q<0.05)
rolling Epi ATR+ vs ATR-	0.04313	0.09102	F
rolling C4da ATR+ vs ATR-	0.04551	0.09102	F
rolling C3da ATR+ vs ATR-	1	1	F
rolling Cho ATR+ vs ATR	1	1	F
c-bend Epi ATR+ vs ATR-	0.4687	0.9508	F
c-bend C4da ATR+ vs ATR-	0.4754	0.9508	F
c-bend C3da ATR+ vs ATR	1	1	F
c-bend Cho ATR+ vs ATR	1	1	F
back Epi ATR+ vs ATR-	0.00000000000004556	0.00000000000018224	T
back C4da ATR+ vs ATR	1	1	F
back C3da ATR+ vs ATR	1	1	F
back Cho ATR+ vs ATR-	0.1128	0.2256	F
hunch Epi ATR+ vs ATR	1	1	F
hunch C4da ATR+ vs ATR	1	1	F
hunch C3da ATR+ vs ATR-	1	1	F
hunch Cho ATR+ vs ATR-	0.4918	1	F
freeze Epi ATR+ vs ATR-	0.00000008682	0.00000034728	T
freeze C4da ATR+ vs ATR-	0.4754	0.6338667	F
freeze C3da ATR+ vs ATR-	1	1	F
freeze Cho ATR+ vs ATR-	0.000825	0.00165	T

Figure 2S1E: Fisher's exact test with BH correction

	p-value	q-value	Significance (q<0.05)
rolling Epi ATR+ vs ATR-	3.039e-06	6.0780e-06	T
rolling C4da ATR+ vs ATR-	9.667e-08	3.8668e-07	T
rolling C3da ATR+ vs ATR-	1	1.0000e+00	F
rolling Cho ATR+ vs ATR-	1	1.0000e+00	F
c-bend Epi ATR+ vs ATR-	1.388e-11	2.776e-11	T
c-bend C4da ATR+ vs ATR-	1.265e-11	2.776e-11	T
c-bend C3da ATR+ vs ATR-	0.00192	2.560e-03	T
c-bend Cho ATR+ vs ATR-	1	1.000e+00	F
back Epi ATR+ vs ATR-	1.238e-13	4.952000e-13	T
back C4da ATR+ vs ATR-	0.6003	6.003000e-01	F
back C3da ATR+ vs ATR-	0.02597	5.194000e-02	F
back Cho ATR+ vs ATR-	0.05251	7.001333e-02	F
hunch Epi ATR+ vs ATR-	0.04313	5.750667e-02	F
hunch C4da ATR+ vs ATR-	0.4754	4.754000e-01	F
hunch C3da ATR+ vs ATR-	1.03e-06	2.060000e-06	T
hunch Cho ATR+ vs ATR-	2.571e-14	1.028400e-13	T
freeze Epi ATR+ vs ATR-	0.01869	0.0747600	F
freeze C4da ATR+ vs ATR-	0.2219	0.2958667	F
freeze C3da ATR+ vs ATR-	0.5353	0.5353000	F
freeze Cho ATR+ vs ATR-	0.05251	0.1050200	F

Figure 3E Fisher's exact test with BH correction

	p-value	q-value	Significance (q<0.05)
rolling control vs *C4da>TNT*	0.001051	0.002102	T
rolling control vs *C3da>TNT*	0.1945	0.194500	F
c-bend control vs *C4da>TNT*	1	1.00000	F
c-bend control vs *C3da>TNT*	0.05219	0.10438	F
back control vs *C4da>TNT*	0.7787	0.778700	F
back control vs *C3da>TNT*	0.005579	0.011158	T
hunch control vs *C4da>TNT*	0.02372	0.04744	T
hunch control vs *C3da>TNT*	0.1124	0.11240	F
freeze control vs *C4da>TNT*	0.3533	0.3533	F
freeze control vs *C3da>TNT*	0.1945	0.3533	F

Figure 3F Kruskal-Wallis test followed by Wilcoxon rank sum test with BH correction

	p-value	q-value	Significance (q<0.05)
Kruskal-Wallis test, rolling	0.0004424		
rolling control vs *C4da>TNT*	0.001122	0.002244	T
rolling control vs *C3da>TNT*	0.7007	0.700700	F
Kruskal-Wallis test, c-bend	0.001948		
c-bend control vs *C4da>TNT*	0.0004278	0.0008556	T
c-bend control vs *C3da>TNT*	0.433	0.4330000	F
Kruskal-Wallis test, back	0.4203		
back control vs *C4da>TNT*	N/A		
back control vs *C3da>TNT*	N/A		
hunch control vs *C4da>TNT*	No Hunch in control		
hunch control vs C3da TNT	No Hunch in control (p=0.4127 C4vsC3)		
Kruskal-Wallis test, freeze	0.3808		
freeze control vs *C4da>TNT*	N/A		
freeze control vs *C3da>TNT*	N/A		

Figure 3S2 D: Fisher's exact test with BH correction

	p-value	q-value	Significance (q<0.05)
Control vs C4da silence, roll	0.0003682	0.0007364	T
Control vs C3da silence, roll	0.3015	0.3015000	F
Control vs C4da silence, c-bend	0.1188	0.2376	F
Control vs C3da silence, c-bend	0.2949	0.2949	F
Control vs C4da silence, backward	1	1.00000	F
Control vs C3da silence, backward	0.02372	0.04744	T
Control vs C4da silence, hunch	1	1	F
Control vs C3da silence, hunch	1	1	F
Control vs C4da silence, freeze	0.1028	0.102800	F
Control vs C3da silence, freeze	0.002466	0.004932	T

Figure 3S2 E: Kruskal-Wallis test followed by Wilcoxon rank sum test

	p-value	q-value	Significance
Kruskal-Wallis test, Roll	0.02471		T
Control vs C4da silence Roll	0.01604	0.03208	T
Control vs C3da silence Roll	0.04658	0.04658	T
Kruskal-Wallis test, c-bend	0.01971		T
Control vs C4da silence c-bend	0.008927	0.017854	T
Control vs C3da silence c-bend	0.02334	0.023340	T

Control vs C4da silence back	0.2222		F
Control vs C3da silence back	N/A		
Control vs C3da silence back	N/A		
Control vs C4da silence hunch	N/A		
Control vs C3da silence hunch	N/A		
Control vs C4da silene freeze	N/A		
Control vs C3da silence freeze	N/A		

Figure 4B: Wilcoxon Rank-Sum test

	p-value	Significance
Fmax/F0 C4da vs C4da +Epi	0.0288	T

Figure 4C: Wilcoxon Rank Sum test

	p-value	q-value	Significance (q<0.05)
C4da vs C4da+Epi	0.03184		T

Figure 4D: Wilcoxon Rank-Sum test

	p-value	Significance
C4da vs C4da+Epi	0.03277	T

Figure 4F: Fisher's exact test with BH correction

	p-value	q-value	Significance (q<0.05)
C4da vs Epi	0.0003136	3.741e-04	T
C4da vs Epi+C4da	0.7541	3.741e-04	T
Epi vs Epi+C4da	0.000005162	2.997e-11	T

Figure 4G: Kruskal-Wallis test followed by Wilcoxon rank sum test with BH correction

	p-value	q-value	Significance (q<0.05)
Kruskal-Wallis test	0.000000001544		
C4da vs Epi	0.7541	0.7541	F
C4da vs Epi+C4da	0.000000000001072	0.000000000003216	T
Epi vs Epi+C4da	0.000005162	0.000007743	T

Figure 4H: Fisher's exact test with BH correction

	p-value	q-value	Significance (q<0.05)
<5 C4da vs Epi	0.1894	1.894e-01	F
<5 C4da vs C4da+Epi	3.646e-11	5.469e-11	T
<5 Epi vs C4da+Epi	2.571e-14	7.713e-14	T
5-9 C4da vs Epi	0.674	0.674	F
5-9 C4da vs C4da+Epi	0.356	0.674	F
5-9 Epi vs C4da+Epi	0.6124	0.674	F
10 -14 C4da vs Epi	0.6144	0.9216	F
10 -14 C4da vs C4da+Epi	1	1.0000	F
10 -14 Epi vs C4da+Epi	0.6124	0.9216	F
15 -19 C4da vs Epi	1	1.0000	F
15-19 C4da vs C4da+Epi	0.1896	0.2844	F
15 -19 Epi vs C4da+Epi	0.1128	0.2844	F
>20 C4da vs Epi	1	1.0000	F
>20 C4da vs C4da+Epi	5.377e-11	1.6131e-10	T
>20 Epi vs C4da+Epi	2.644e-10	3.9660e-10	T

Figure 4I: Kruskal-Wallis test followed by Wilcoxon rank sum test with BH correction

	p-value	q-value	Significance (q<0.05)
Kruskal-Wallis test	5.993e-15		
C4da vs Epi	0.0009667	9.6670e-04	T
C4da vs Epi+C4da	4.97e-15	7.4550e-15	T
Epi vs Epi+C4da	5.028e-16	1.5084e-15	T

Figure 4J: Kruskal-Wallis test followed by Wilcoxon rank sum test with BH correction

	p-value	q-value	Significance (q<0.05)
Kruskal-Wallis test, rolling	4.024e-09		T
roll C4da vs Epi	0.1937	1.9370e-01	F
roll C4da vs C4da+Epi	9.024e-12	2.7072e-11	T
roll Epi vs C4da + Epi	5.506e-05	8.2590e-05	T
Kruskal-Wallis test, c-bend	0.06175		F
c-bend C4da vs Epi	N/A		
c-bend C4da vs C4da+Epi	N/A		
c-bend Epi vs C4da +Epi	N/A		
Kruskal-Wallis test, backing	9.145e-06		T
back C4da vs Epi	9.574e-07	2.8722e-06	T
back C4da vs C4da+Epi	0.02662	2.6620e-02	T
back Epi vs C4da+Epi	0.001987	2.9805e-03	T
Kruskal-Wallis test, hunching	0.006437		T
hunch C4da vs Epi	0.2706	0.270600	F
hunch C4da vs C4da+Epi	0.001252	0.003756	T
hunch Epi vs C4da+Epi	0.1836	0.270600	F
Kruskal-Wallis test, freezing	0.4857		F
freeze C4da vs Epi	N/A		
freeze C4da vs C4da+Epi	N/A		
freeze Epi vs C4da+Epi	N/A		

Figure 4K: Fisher's exact test with a BH correction

Epi-GAL4, UAS-CsChrimson treatment groups	p-value	q-value	Significance
20 mN ATR- vs ATR+ Epi>CsChrimson	0.003234	0.0097020	T
50 mN ATR- vs ATR+ Epi>CsChrimson	0.008797	0.0131955	T
50mN ATR- vs ATR+ CsChrimson	0.7639	0.7639000	F

Figure 4L: Fisher's exact test with BH correction

	p-value	q-value	Significance (q<0.05)
No *GAL4* 25 vs 32	0.6798	0.7806000	F
Epi-GAL4 25 vs 32	0.0005454	0.0021816	T
27H06-GAL4 25 vs 32	0.7806	0.7806000	F
ppk-GAL4 25 vs 32	0.7735	0.7806000	F

Figure 4M: Fisher's exact test with BH correction

UAS-TRPA1 comparisons	p-value	q-value	Significance (q<0.05)
10sec control vs 10sec *Epi-GAL4*	0.006108	0.01018000	T
30sec control vs 30sec *Epi-GAL4*	0.001265	0.00316250	T
60sec control vs 60sec *Epi-GAL4*	0.00007129	0.00035645	T
300sec control vs 300sec *Epi-GAL4*	0.07531	0.09413750	F
600sec control vs 600sec *Epi-GAL4*	0.4351	0.43510000	F

Figure 4N: Curve fitting

	fit curve	Decay time constant
Mechano (Fig 4S1B)	f(x) = 0.4139*exp(-0.002994*x)	334.001336
Epidermal activation (Fig 4L)	f(x) = 0.47*exp(-0.002964*x)	337.3819163

Figure 4S1B: Fisher's exact test with BH correction

	p-value	q-value	Significance (q<0.05)
10sec 1st vs 2nd	0.0007546	0.0018865	T
30sec 1st vs 2nd	0.000006865	0.000034325	T
60sec 1st vs 2nd	0.008544	0.01424	T
300sec 1st vs 2nd	0.131	0.16375	F
600sec 1st vs 2nd	0.3217	0.3217	F

Figure 4S1C: Fisher's exact test with BH correction

	p-value	q-value	Significance (q<0.1)
Control 1st vs 2nd	0.006746	0.00674600	T
Epi-GAL4 1st vs 2nd	6.336e-05	0.00019008	T
Nociceptor-GAL4 1st vs 2nd	0.005186	0.00674600	T

Figure 6B Fisher's exact test with BH correction

	p-value	q-value	Significance (q<0.1)
Control 1st vs 2nd	4.94E-05	0.0002472	T
Orai RNAi 1st vs 2nd	0.1329	0.3236667	F
Stim RNAi 1st vs 2nd	0.1942	0.3236667	F
Control 1st vs *Orai* RNAi 1st	0.8788	0.6660000	F
Control 1st vs *Stim* RNAi 1st	0.5328	0.8788000	F

Figure 6E: Chi-square comparing distribution of stretch sensitive cells

	Chi-square value	DF	p-value
Control vs *Stim* RNAi	33	4	<0.0001
Control vs *Orai* RNAi	150.6	4	<0.0001
Stim RNAi vs *Orai* RNAi	3.689	4	0.4497

Figure 6G: Chi-square comparing distribution of stretch sensitive cells

	Chi-square value	DF	p-value
Control vs La3+	36.09	4	<0.0001
Control vs Store Depleted	112.1	4	<0.0001
Store Depleted vs La3+	40.69	4	<0.0001

Figure 6J: Fisher's exact test

	p-value		
control vs *GtACR*	0.03216		

Figure 6K Fisher's exact test with BH correction

	p-value	q-value	Significance (q<0.1)
Control 1st vs 2nd	0.00037	0.00148000	T
Stim OE 1st vs 2nd	0.02386	0.03181333	T
Control 1st vs *Stim OE* 1st	0.002115	0.00423000	T

Figure 6L: Fisher's exact test with BH correction

	p-value	q-value	Significance
Control 25C 1st vs Control 25C 2nd	5.425e-05	7.233333e-05	T
Control 30C 1st vs Control 30C 2nd	1.033e-06	2.066000e-06	T
Epi>shi 25C 1st vs *Epi>shi* 25C 2nd	4.912e-07	1.964800e-06	T
Epi>shi 30C 1st vs *Epi>shi* 30C 2nd	0.05076	5.076000e-02	F

Figure 6S2A Fisher's exact test with BH correction

	p-value	q-value	significance
Control Trial 1 vs Trial 2	0.002438	0.004876	T
Orai RNAi Trial 1 vs Trial 2	0.4076	0.4076	F

Figure 6S2B Fisher's exact test with BH correction

	p-value	q-value	significance
Control Trial 1 vs Trial 2	0.00157	0.003140	T
Task6 RNAi Trial 1 vs Trial 2	0.004795	0.004795	T

Figure 6S2C Wilcoxon Rank-Sum test

	p-value	significance
Control vs La^{3+} block	0.0003377	T

Figure 6S2E: Kruskal-Wallis test followed by Wilcoxon rank sum test with BH correction

	p-value	q-value	Significance (q<0.05)
Kruskal-Wallis test	0.00001332		T
Control vs *Stim* RNAi	0.0009161	0.001374150	T
Control vs *Orai* RNAi	0.000005094	0.000015282	T
Stim RNAi vs *Orai* RNAi	0.3759	0.375900000	F

Figure 6S2F: Kruskal-Wallis test followed by Wilcoxon rank sum test with BH correction

	p-value	q-value	Significance (q<0.05)
Kruskal-Wallis test	0.004105		T
Control vs *Stim* RNAi	0.001296	0.002592	T
Control vs *Orai* RNAi	0.8294	0.829400	F
