# Supplementary material for: Drosophila epidermal cells are intrinsically mechanosensitive and modulate nociceptive behavioral outputs
Source: eLife. 2025 May 12;13:RP95379. doi: 10.7554/eLife.95379 (PMC12068870; doi:10.7554/eLife.95379)
Supplement: Supplementary file 2. [file elife-95379-supp2.pdf]

Supplementary File 2. Larval behaviors scored in this study

**Behavior**	**Description / Criteria**
Locomotion	Continuous forward movement with smooth, regular peristalsis
Pausing	Break in locomotion, lasting less than 1 second
Freezing	Break in locomotion that persists for longer than 1 second
Hunching	Contraction along the anterior-posterior axis; no forward / backward motion or curling
Backward locomotion	Anterior-to-posterior peristalsis accompanied by movement in reverse
Writhing crawl	Forward movement with anterior and posterior sweeping of the body, tilting onto the side axis
C-bending	Bending into a c-shape, with both head and tail bending in the same direction; includes incomplete rolls
Rearing	Upwards bending or arching of anterior part of the body, with posterior part largely stationary
Rolling	Larva completes a full 360-degree rotation of its body
