# Supplementary material for: Drosophila epidermal cells are intrinsically mechanosensitive and modulate nociceptive behavioral outputs
Source: eLife. 2025 May 12;13:RP95379. doi: 10.7554/eLife.95379 (PMC12068870; doi:10.7554/eLife.95379)
Supplement: Supplementary file 3. [file elife-95379-supp3.pdf]

Supplementary File 3. Solutions used in calcium imaging studies.

**HL3.1, *Drosophila* saline**		
**Compound**	**M.W. (g/mol)**	**1X Concentration (mM)**
NaCl	58.44	120
KCl	74.55	5
Proline	115.13	5
HEPES	238.30	10
Trehalose	378.33	5
Sucrose	342.30	32.5
$CaCl_2$	1M stock	1.5
$MgCl_2$	1M stock	1
pH (with NaOH)	7.15	
Osmolarity (mOsm/L)	310	

**Zero Ca^{2+} / EGTA HL3.1**		
**Compound**	**M.W. (g/mol)**	**1X Concentration (mM)**
NaCl	58.44	120
KCl	74.55	5
Proline	115.13	5
HEPES	238.30	10
Trehalose	378.33	5
Sucrose	342.30	32.5
EGTA	380.35	1.5
$MgCl_2$	1M stock	1
pH (with NaOH)	7.15	
Osmolarity (mOsm/L)	310	

**20mM Ca HL3.1**		
**Compound**	**M.W. (g/mol)**	**1X Concentration (mM)**
NaCl	58.44	101.5
KCl	74.55	5
Proline	115.13	5
HEPES	238.30	10
Trehalose	378.33	5
Sucrose	342.30	32.5
$CaCl_2$	1M stock	20
$MgCl_2$	1M stock	1
pH (with NaOH)	7.15	
Osmolarity (mOsm/L)	310	

**Isotonic** (modified from *Drosophila* saline to maintain ionic balance across osmolarities)		
**Compound**	**M.W. (g/mol)**	**1X Concentration (mM)**
NaCl	58.44	90
KCl	74.55	5
Proline	115.13	5
HEPES	238.30	10
Trehalose	378.33	5
Sucrose	342.30	92.5
$CaCl_2$	1M stock	1.5
$MgCl_2$	1M stock	1
pH (with NaOH)	7.15	
Osmolarity (mOsm/L)	310	

85% Hypoosmotic		
**Compound**	**M.W. (g/mol)**	**1X Concentration (mM)**
NaCl	58.44	90
KCl	74.55	5
Proline	115.13	5
HEPES	238.30	10
Trehalose	378.33	5
Sucrose	342.30	46.5
$CaCl_2$	1M stock	1.5
$MgCl_2$	1M stock	1
pH (with NaOH)	7.15	
Osmolarity (mOsm/L)	264	

70% Hypoosmotic		
**Compound**	**M.W. (g/mol)**	**1X Concentration (mM)**
NaCl	58.44	90
KCl	74.55	5
Proline	115.13	5
HEPES	238.30	10
Trehalose	378.33	5
Sucrose	342.30	0
$CaCl_2$	1M stock	1.5
$MgCl_2$	1M stock	1
pH (with NaOH)	7.15	
Osmolarity (mOsm/L)	217.5	
